# The poor prognosis of lacrimal gland adenocarcinoma: a clinical study and literature review

**DOI:** 10.1007/s00432-023-05510-7

**Published:** 2024-01-23

**Authors:** Rui Liu, Tingting Ren, Jing Li, Nan Wang, Liangyuan Xu, Qihan Guo, Hong Zhang, Jianmin Ma

**Affiliations:** 1 Beijing Institute of Ophthalmology, Beijing Tongren Eye Center, Beijing Tongren Hospital, Capital Medical University, Beijing, 100730 China; 2 Pathology, Beijing Tongren Hospital, Capital Medical University, Beijing, 100730 China

**Keywords:** Adenocarcinoma, Ductal adenocarcinoma, Lacrimal gland, Prognosis, Treatment

## Abstract

**Purpose:**

The incidence of lacrimal gland adenocarcinoma is low. This study was designed to analyze the clinical and prognostic characteristics of lacrimal gland adenocarcinoma.

**Methods:**

This was a clinical study and literature review; 25 patients diagnosed with lacrimal gland adenocarcinoma by histopathology were enrolled and their medical history data were collected.

**Results:**

The incidence of bone destruction and surrounding tissue invasion was 52% and 44%, respectively. The incidence of distant metastasis of lacrimal gland adenocarcinoma was about 50%. The 5-year overall survival rate of death or metastasis was 33.5%. Age, sex, laterality, tumor size, pathology type, bone destruction, nerve or perineural invasion, invasion of peripheral tissue, T stage, AR, Her-2 and treatment had no significant correlation with lacrimal adenocarcinoma’s prognosis (*P* > 0.05), while the higher expression of Ki-67 may have higher risk of death or metastasis (*P* = 0.020).

**Conclusion:**

The incidence of bone destruction and distant metastasis of lacrimal adenocarcinoma is high and the imaging examination is necessary to assess the risk of distant metastasis. The 5-year survival rate of death or metastasis is 33.5% and the high expression of Ki-67 predicts poor prognosis of lacrimal adenocarcinoma.

**Supplementary Information:**

The online version contains supplementary material available at 10.1007/s00432-023-05510-7.

## Introduction

Adenocarcinoma of the lacrimal gland is a malignant epithelial tumor of low incidence. Ashok et al. analyzed 669 cases of lacrimal gland tumor; adenocarcinoma accounted for 10.46% of them (Ashok Kumar et al. 2022). Andreoli et al. analyzed 702 cases of malignant lacrimal gland tumor; adenocarcinoma accounted for 3.8% of the cases (Andreoli et al. 2015). A statistical analysis of 281 malignant primary orbital tumors by Goto et al. showed that the incidence of adenocarcinoma was only 3% (Goto et al. 2021). Primary adenocarcinoma is very rare, accounting for only 5–7% of epithelial tumors of the lacrimal gland (Touil et al. 2017). Primary ductal adenocarcinoma (PDA) was classified under adenocarcinoma of the lacrimal gland in the World Health Organization Classification of Tumors (Alkatan et al. 2018). Adenocarcinoma is an aggressive lacrimal gland malignancy whose pathogenesis remains unclear. Studies have shown that androgen receptors are expressed in lacrimal adenocarcinoma; thus, androgen receptor expression and deprivation combined with checkpoint inhibition may be effective in the treatment of lacrimal adenocarcinoma (Bulbul 2018). Andreasen et al. (2017) believe that the genetic characteristics and protein expression pattern of this malignancy are similar to those of ductal adenocarcinoma of the breast and salivary glands; they, therefore, suggest that HER2 gene amplification, PTEN, CDKN2A, and HRAS gene aberrations may be related to its pathogenesis. We totally collected 25 cases with lacrimal gland adenocarcinoma to evaluate their clinical and prognostic characteristics.

## Materials and methods

### Objects

Twenty-five patients admitted to the Ophthalmic Oncology Department of Beijing Tongren Hospital, Capital Medical University, from October 2013 to October 2023 were selected for this study. 22 patients underwent surgery and histopathological examination in our hospital, and 3 patients underwent ^125^I radiotherapy in our hospital after surgery in other hospitals. All patients were supplemented with missing information after follow-up. Ours was a single-center clinical study and the incidence of lacrimal adenocarcinoma is low; thus the number of cases examined is small. Inclusion criteria: (1) Lacrimal gland adenocarcinoma confirmed by histopathology and immunohistochemical staining; (2) Availability of a complete medical history included age, gender, laterality, clinical manifestations or prognosis, etc. Exclusion criteria: (1) Presence of other malignant lacrimal gland tumors; (2) Incomplete medical history; (3) Presence of a metastatic tumor and a history of other systemic malignancies. All subjects were fully aware of the purpose of the study and informed consent was obtained. The study was supported by the ethics committee of Beijing Tongren Hospital, Capital Medical University, in accordance with the principles of the Declaration of Helsinki.

### Data analysis and laboratory methods

Basic information included gender, age, laterality, clinical manifestations, human epidermal growth factor receptor 2 (Her-2), androgen receptor (AR), estrogen receptor (ER), Ki-67, TNM stage, treatment methods, follow-up time, and prognosis were collected. The surgery of orbital malignant tumors mainly includes tumor resection (TR) and exenteration (ET) when necessary. Adjuvant treatment modalities include radiotherapy (RT) (external radiotherapy and ^125^I internal radiotherapy), chemotherapy (CT), and targeted drug therapy. Chemotherapy was performed at an external treatment center, and the method and dosage of treatment was unknown. ^125^I seeds could be implanted at the inner, outer, superior or lower margins of the superior temporal muscle, and the superior and lower margins of the external rectus muscle, with an interval of 1 cm and a distance of 0.8 cm from the skin. The average number of implanted seeds was 20, with an activity of 0.7–0.9 mCi per seed. The field for γ-ray therapy included the superior and inferior orbital fissures and the anterior cavernous or skull base depending on the areas of tumor invasion. The cumulative radiation dose was approximately 60–70 Gy/6 w–7 w, and the single radiation dose was 180–200 cGy. Imaging examination, intraoperative evaluation or histopathologic examination showed bone invasion, nerve invasion, invasion of other tissue and metastasis. Recurrence or metastasis was confirmed by imaging examination or histopathologic examination.

### Statistical analysis

SPSS 25.0 (SPSS Inc., Chicago, IL) and GraphPad Prism 8.0 (GraphPad Software Inc., La Jolla, CA) software were used to process the data. The Kolmogorov–Smirnov test was used to test the normality of the data. Measurement data conforming to a normal distribution were expressed as mean ± SD, and comparison between groups was performed using the *t* test. Measurement data conforming to nonnormal distributions were expressed as median, and comparisons between groups were performed using the Mann–Whitney or Kruskal–Wallis test. The count data were analyzed using the chi-squared test or Fisher’s exact test. With death or metastasis as the end point, survival analysis was performed using Log-rank test. The follow-up period began with the patient's first treatment at our hospital and ended with the last follow-up time or the patient's death or metastasis. *P* < 0.05 was considered to be statistically significant.

## Results

### Baseline features of patients

Overall, a total of 25 patients have been studied, including 19 males and 6 females, with a male-to-female ratio of 3.2:1. Their average age was 56.20 ± 11.58 years (range 33–77 years). There were 16 cases involving the left eye and 9 involving the right. The main clinical manifestations were exophthalmos in 13 cases (52%), ocular mass in 9 (36%), and eyelid swelling in 6 (24%). Secondary manifestations included ocular pain in 5 cases (20%), eye movement disorder in 3 (12%), ptosis in 2 (8%), visual impairment in 1 (4%), and diplopia in 1 (4%). The average tumor size was 3.04 ± 0.87 cm.

### Imaging and pathological findings

**Fig. 1:**
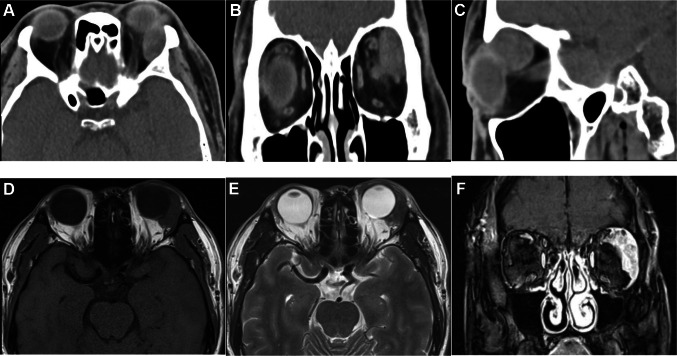
Imaging findings of lacrimal duct adenocarcinoma. **A** The lacrimal gland mass of the left eye had clear boundaries and uneven density. **B** The left lacrimal gland mass compressed the eyeball. **C** The left lacrimal gland mass compressed the extraocular muscle and bone wall. **D** A mass in the left lacrimal gland with blurred boundaries and indistinct boundaries from surrounding tissue, and a medium T1-weighted images. **E** A slightly hypointense signal on T2-weighted images. **F** The tumor showed obvious enhancement on enhanced scan

The pathological types included 3 cases of primary adenocarcinoma (NOS) (12%), 3 cases of adenocarcinoma ex pleomorphic adenoma (12%), and 19 cases of ductal adenocarcinoma (76%). Bone destruction occurred in 13 patients (52%), peripheral tissue invasion in 11 cases (44%), and optic nerve or perineural invasion in 5 cases (20%). The main sites of bone destruction were the supraorbital wall and lateral orbital wall, other sites included medial orbital wall, inferior orbital wall, great wing of sphenoid, sieve interatrial septum, sphenoid sinus, sphenoid bone, basilar clivus, and frontal bone. The main sites of peripheral tissue invasion were the adipose tissue, levator palpebrae muscle, the superior rectus, and the eyelid. Computed tomography examination showed lacrimal gland mass with clear boundary and uneven density, and the eyeball could be slightly protruded (Fig. 1A–C). Magnetic resonance imaging examination showed that the tumor had slightly longer T1 and T2 signals, which could involve the surrounding tissues (Fig. 1D, E). The enhanced scan showed obvious enhancement (Fig. 1F), and the dynamic enhancement curve showed rapid rising and outflow type.

**Fig. 2:**
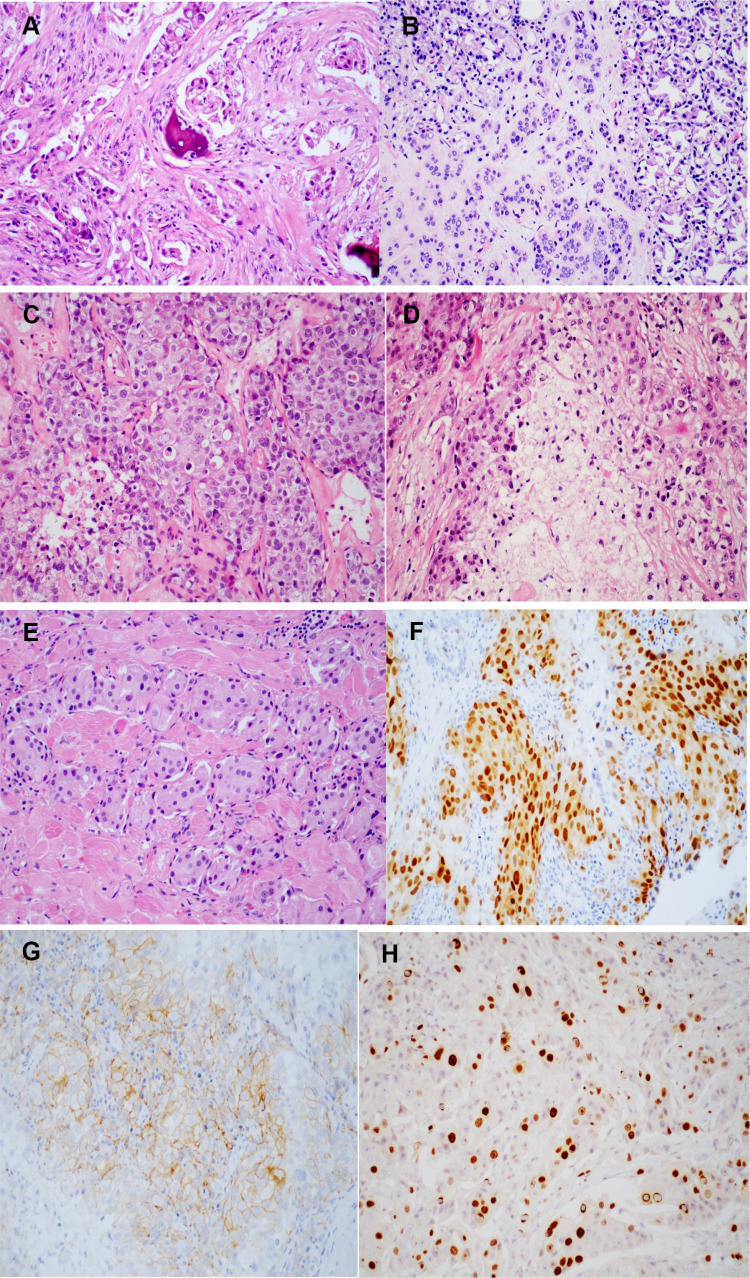
Histopathologic findings of adenocarcinoma of lacrimal gland. **A** Bone tissue was invaded by tumor cells; **B** tumor cells invaded lacrimal gland; **C** lacrimal gland ductal carcinoma; **D** tumor necrosis; **E** the tumor invaded the striated muscle tissue; **F** the positive expression of AR. **G** The positive expression of Her-2; **H** the positive expression of Ki-67

Positive expression of Her-2 in 10 cases, negative in 4 cases, and unknown in 11 cases. Positive expression of AR in 17 cases, negative in 4 cases, and unknown in 4 cases. Negative expression of ER in 4 cases and unknown in 21 cases. The expression level of Ki-67 was 32.60 ± 24.58 (%). The histopathological findings of the tumor were shown in Fig. 2. Of 18 patients in the T1/T2 stage, 9 (50%) died or had metastasis. There were 7 patients in the T3/T4 stage, of whom 5 (71.4%) died or had metastasis.

### Prognosis and factors influencing mortality

TR + RT was performed in two patients and no one died or had metastasis. TR + ^125^I was performed in 17 cases, of whom 10 (58.8%) died or had metastasis. Three patients underwent ET; two (66.7%) of these died or developed metastasis. Three patients underwent TR + RT + CT; two (66.7%) of these died or developed metastasis. The mean follow-up time was 32.88 ± 21.99 months (range 5–90 months). A total of 11 (44%) patients remained alive without disease (AOD), 2 (8%) remained alive with disease (AWD), 2 (8%) died from other causes (DOC), and 10 (40%) died with disease (DWD). There were 12 (50%) cases of distant metastasis and one case was unknown: 9 (75%) of the brain, 3 (25%) of the lymph node, 2 (16.7%) of the lung, and 2 (16.7%) of the parotid gland.

**Fig. 3:**
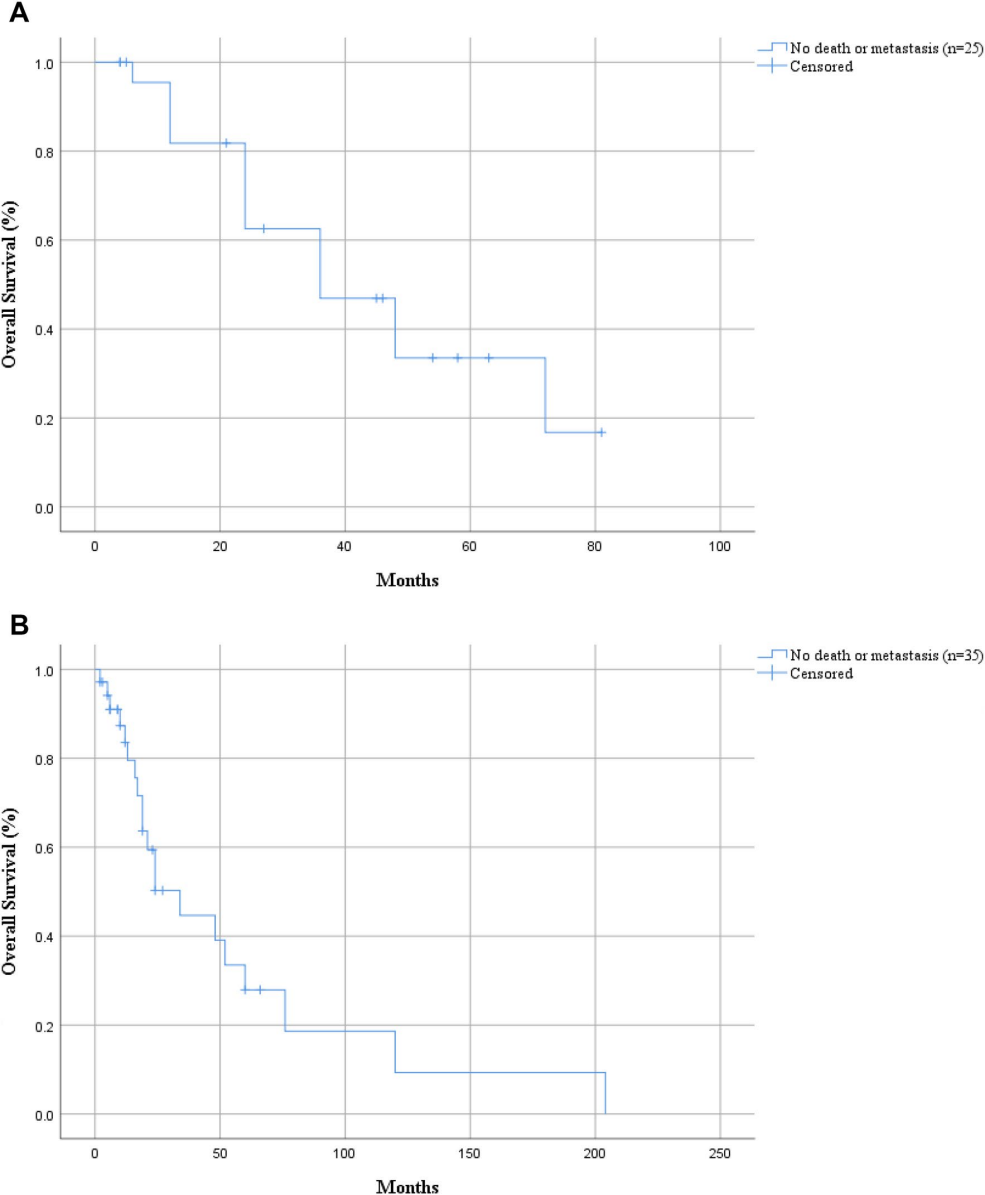
Overall survival analysis of adenocarcinoma of the lacrimal gland. **A** The 5-year survival rate of death or metastasis in 25 our cases is 33.5%. **B** The 5-year survival rate of death or metastasis in 35 cases of literature reported is 27.9%

The 5-year overall survival rate of death or metastasis for the total 25 patients was 33.5% (Fig. 3A). Overall analysis showed that age, sex, laterality, tumor size, pathology type, bone destruction, nerve or perineural invasion, invasion of peripheral tissue, T stage, AR, Her-2, and treatment had no significant correlation with prognosis (*P* > 0.05), while the higher expression of Ki-67 may have higher risk of death or metastasis (*P* = 0.020) (Table 1).

**Table 1:** Univariate analysis of factors affecting death or metastasis in our 25 cases

Characteristic	Death or metastasis		Test value	*P*
	Yes	No		
Gender (male/female)	10/4	9/2	–	0.661^b^
Mean age (years)	57.64 ± 13.05	54.36 ± 9.67	− 0.695	0.494^a^
Laterality				
Left	9	7	–	1.000^b^
Right	5	4		
Bone destruction	8	5	–	0.695^b^
Nerve or perineural invasion	4	1	–	0.341^b^
Peripheral tissue invasion	6	5	–	1.000^b^
Tumor size (cm)	3.29 ± 0.94	2.72 ± 0.70	− 1.693	0.104^a^
Pathology type				
Adenocarcinoma (NOS)	1	2	0.963	0.813^b^
Adenocarcinoma ex pleomorphic adenoma	2	1		
Ductal adenocarcinoma	11	8		
AR+	9	8	–	0.603^b^
Her-2+	6	4	–	1.000^b^
Ki-67 (%)	42.50 ± 25.78	20.00 ± 16.58	− 2.510	**0.020**^a^
T stage				
T1–T2	9	9	–	0.407^b^
T3–T4	5	2		
Treatment				
ET	2	1	2.659	0.548^b^
TR + RT	0	2		
TR + RT + CT	2	1		
TR + ^125^I	10	7		
Follow-up time	29.57 ± 18.00	37.09 ± 26.54	0.844	0.408^a^

Bold value indicates *P* < 0.05

^a^*t* test

^b^Fisher’s exact test

## Discussion

Adenocarcinoma is a rare but aggressive lacrimal gland malignancy whose pathogenesis remains unclear. The disease was the most common in the male patients and the incidence of bone destruction and surrounding tissue invasion was 52% and 44%, respectively. But no significant correlation between bone destruction or peripheral tissue invasion and the prognosis was found in this research. Imaging findings showed malignant signs, including an irregularly shaped mass, bone destruction (invasion or compression of the tumor), and calcification (Yang et al. 2023). Magnetic resonance imaging examination showed that the tumor had isointense internal signal on T1-weighted images, a hyperintense signal on T2-weighted images, and moderate contrast enhancement (Gündüz et al. 2003). Rapid rising and outflow curve was helpful in differentiating benign and malignant lesions.

The incidence of distant metastasis of lacrimal gland adenocarcinoma was about 50% in this study. Due to the special location of lacrimal gland, the incidence of brain metastasis and cervical lymph node metastasis was higher, 75% and 25%, respectively. Therefore, attention should be paid to brain imaging and cervical lymph node examination during diagnosis, and whole-body PET-CT examination should be performed if necessary.

Histopathologic examination is the main criterion for definite diagnosis. The main pathologic manifestations are polygonal tumor cells; granular eosinophilic cytoplasm; pleomorphic nuclei, nucleoli, and mitosis; and central comedo-like necrosis (Yang et al. 2023). There was no significant difference in prognosis between primary adenocarcinoma (NOS), adenocarcinoma ex pleomorphic adenoma and primary ductal adenocarcinoma. Immunohistochemical characteristics of AR, CK7, GCDFP-15, and EMA are usually positive, PR, ER, and PSA are usually negative, and the Ki-67 proliferation index may reach 60% (See et al. 2020; Zhu et al. 2015; Kubota et al. 2013). Overexpression of ERBB2 (HER2) and p53 is associated with a poor prognosis, whereas GCDFP-15, AR, and EBBR2 have been proposed as treatment guidelines and prognostic biomarkers (See et al. 2020; Zhu et al. 2015; Katz et al. 1996). Positive expression of AR plays an important guiding role in the diagnosis of ductal adenocarcinoma. However, the positive expression rate of AR in salivary duct carcinoma can reach 56–97.8% and in breast cancer can reach 60–90% (Udager and Chiosea 2017; Ravaioli et al. 2022). In our study, the positive expression rate of AR in lacrimal duct adenocarcinoma was about 81%. AR is also an important prognostic indicator and studies have shown that ductal carcinoma in situ of the breast with negative AR expression has a worse prognosis and a higher recurrence rate (Lee et al. 2020; Anestis et al. 2020). The histological features of ductal carcinoma of the salivary and lacrimal glands are similar to those of the breast (Luna-Ortiz et al. 2022). Due to the low incidence of salivary gland and lacrimal duct carcinoma, we did not find relevant literature reporting the correlation between AR expression and prognosis. And no significant correlation was found between the expression of Her-2 and the prognosis. Ki-67 is an indicator of tumor activity and the higher expression of Ki-67 cases have higher risk of death or metastasis in this study.

The prognosis of adenocarcinoma of the lacrimal gland is poor and the prognosis of patients in T1/2 stage is as poor as that in T3/4 stage. Studies have shown a 5-year survival rate of 40–44% and the 10-year disease-specific and overall survival rates were 51.4% and 27.7%, respectively (Andreoli et al. 2015; Kubota et al. 2013). We took death or metastasis as the endpoint and found that the 5-year overall survival rate for the total 25 patients was 33.5%. We conducted survival analyses of ET, TR + ^125^I, TR + RT, and TR + RT + CT and found no significant difference. At present, there is no unified standard of treatment, and the choice of treatment depends on the disease characteristics, including tumor size, location, malignancy degree, scope of involvement, and distant metastasis.

Due to the small number of lacrimal adenocarcinoma cases, we also reviewed the literature in the PubMed that had been published about adenocarcinoma or duct adenocarcinoma of the lacrimal gland. The search keywords were “adenocarcinoma”, “duct adenocarcinoma”, and “lacrimal gland”. 35 lacrimal gland adenocarcinoma cases published from 2003 to 2023 with relatively complete data had been enrolled (Touil et al. 2017; See et al. 2020; Zhu et al. 2015; Kubota et al. 2013; Katz et al. 1996; Yang et al. 2018; Patel et al. 2018; Dennie 2015; Lau et al. 2015; Ricci et al. 2014; Min et al. 2014; Damasceno and Holbach 2012; Lee and Oh 2009; Kim et al. 2008, 2022; Milman et al. 2005; Kurisu et al. 2005; Krishnakumar et al. 2003; Fakhril-Din et al. 2023; Issiaka et al. 2021; Ashok Kumar et al. 2020; Garakani et al. 2020; Aucoin et al. 2023). We found the incidence of bone destruction and surrounding tissue invasion was 51.5% and 30.3%, respectively. The 5-year survival rate of death or metastasis in 35 cases of literature reported is 27.9% (Fig. 3B). Age, sex, laterality, tumor size, pathology type, bone destruction, nerve or perineural invasion, invasion of peripheral tissue, T stage, AR, Her-2, and Ki-67 had no significant correlation with prognosis (*P* > 0.05) (Table 2). The reason for the lack of correlation between Ki-67 and prognosis may be related to the different treatment methods. Our analysis showed that the prognosis of ET + RT (11 cases) was better than that of ET (4 cases), and the prognosis of TR + RT (5 cases) was better than that of TR + CT (2 cases). However, this result needs to be verified due to the small sample size.

**Table 2:** Univariate analysis of factors affecting death or metastasis in 35 cases of literature reported

Characteristic	Death or metastasis		Test value	*P*
	Yes	No		
Gender (male/female)	19/1	10/5	–	0.064^b^
Mean age (years)	60.55 ± 11.51	58.07 ± 13.18	− 0.594	0.557^a^
Laterality				
Left	9	9	–	0.500^b^
Right	11	6		
Bone destruction	11	6	–	0.491^b^
Nerve or perineural invasion	1	3	–	0.288^b^
Peripheral tissue invasion	6	4	–	1.000^b^
Tumor size (cm)	3.27 ± 1.28	2.84 ± 1.21	− 0.980	0.335^a^
Pathology type				
Adenocarcinoma (NOS)	3	1	1.248	0.574^b^
Adenocarcinoma ex pleomorphic adenoma	1	2		
Ductal adenocarcinoma	16	12		
AR+	13	8	–	1.000^b^
Her-2+	11	7	–	1.000^b^
Ki-67 (%)	37.75 ± 21.66	57.50 ± 28.72	1.344	0.209^a^
T stage				
T1–T2	9	9	–	0.482^b^
T3–T4	10	5		
Treatment				
ET	4	0	11.451	**0.042**^**b**^
TR + RT	2	3		
TR + RT + CT	5	2		
ET + RT	7	4		
TR + CT	1	1		
TR	0	5		
RT	1	0		
Follow-up time	39.10 ± 48.30	18.73 ± 19.65	-1.536	0.134^a^

Bold value indicates *P* < 0.05

^a^*t* test

^b^Fisher's exact test

This study has some limitations. The number of study cases is small, which may lead to differences in study results and imperfect studies on prognostic factors. We report a single-center study with the largest number of cases and analyze the clinical prognostic characteristics of lacrimal adenocarcinoma in conjunction with the literature. The results show that the incidence of bone destruction and distant metastasis of lacrimal adenocarcinoma is high and the imaging examination is necessary to assess the risk of distant metastasis. The 5-year survival rate of death or metastasis is 33.5% and the high expression of Ki-67 predicts poor prognosis of lacrimal adenocarcinoma.

## Supplementary Information

Below is the link to the electronic supplementary material.

Supplementary file1 (XLSX 18 KB)

## Data Availability

Not applicable.

## Abbreviations

Her-2: Human epidermal growth factor receptor 2
AR: Androgen receptor
ER: Estrogen receptor
ET: Exenteration
TR: Tumor resection
RT: Radiotherapy
CT: Chemotherapy
AOD: Alive without disease
AWD: Alive with disease
DOC: Dead of other cause
DWD: Disease-related death
